# Factors Affecting Secondhand Smoke Avoidance Behavior of Vietnamese Adolescents

**DOI:** 10.3390/ijerph15081632

**Published:** 2018-08-02

**Authors:** Ja-yin Lee, Hyunmi Ahn, Hyeonkyeong Lee

**Affiliations:** 1 College of Nursing, Yonsei University, Seoul 03722, Korea; angela_1004@hanmail.net; 2 Department of Nursing, Korean Bible University, Seoul 01757, Korea; esderahn@bible.ac.kr or esderahn@gmail.com; 3 College of Nursing, Mo-Im Kim Nursing Research Institute, Yonsei University, Seoul 03722, Korea; HLEE39@yuhs.ac

**Keywords:** secondhand smoke, avoidance behavior, adolescent, Vietnam

## Abstract

The purpose of this study was to examine the stage of secondhand smoke avoidance behavior of adolescents in rural areas of Vietnam and the related factors affecting such behavior. The participants were recruited from two middle schools located in Hai Thuong and Trieu Trach commune, Vietnam. Data were collected in January 2016 by distributing and collecting a self-administrated questionnaire. The collected data were analyzed (n = 166) using SPSS 21.0 for frequency, percentage, and ordinal logistic regression. The largest proportion of secondhand smoke avoidance behavior was classified in maintenance (41.6%), followed by action (19.9%), preparation (15.1%), pre-contemplation (13.3%), and contemplation (10.2%) stages. The factors related to higher secondhand smoke avoidance behavior were higher self-efficacy (*p* = 0.003) and more frequent discussion with parents about dangers of smoking (*p* = 0.001). The findings indicated that self-efficacy of avoiding secondhand smoke and discussion with parents were associated with secondhand smoke avoidance behavior of adolescents. These findings can be used for developing education programs to prevent secondhand smoke for adolescents in Vietnam.

## 1. Introduction

Secondhand smoking (SHS), also known as environmental tobacco smoking, is the inhalation of smoke by a nonsmoker, released from the burning tobacco or exhaled by the smoker. Adults exposed to SHS are more likely to develop diseases such as cardiovascular diseases and gastric cancer, and children are at a higher risk of acute respiratory infections, asthma exacerbations, and sudden infant death syndrome, among other conditions [1]. Therefore, SHS is considered to have a negative effect on health. In Vietnam, 23.8% of adults over the age of 15 are smokers, making it one of the countries with the highest smoking rate [2]. With regard to gender, the smoking rate in Vietnam was found to be significantly higher in males (47.4%) than in females (1.4%), but there was no significant difference between rural (24%) and urban areas (23.3%). On the other hand, the total rate of SHS among adults within households was found to be 73.1%, and the rate was higher in rural areas (77.4%) compared to urban areas (63.3%) [2]. The smoking rate among adolescents aged 13–15 years was found to be low at 2.3%, but the percentage experiencing SHS during the week was 73.7%; thus, the majority of adolescents were found to be exposed to SHS [3].

Avoidance behavior (such as avoiding places where one is exposed to secondhand smoke or opening the window to ventilate the room to remove contaminated air) is an effective way to prevent SHS in adolescents, as it greatly reduces exposure to tobacco smoke. According to previous studies, there was a higher tendency to avoid SHS among individuals who had no experience of smoking or who had a willingness to quit [4]. In addition, SHS avoidance behavior was directly influenced by self-efficacy in this behavior [5]. This study defined self-efficacy in avoidance behavior as the degree of confidence in completing the specific task at hand. According to a study by Huang et al. [6], before smoking indoors was prohibited by the government, self-efficacy in SHS avoidance behavior among children aged 8–13 was significantly correlated with gender, exposure to SHS at home, and attitude towards smoking. After the enactment of the law, the attitude towards smoking and knowledge of tobacco hazards were found to be influential. In a study conducted with adults, it was found that a higher knowledge of SHS was associated with a more positive attitude towards SHS avoidance behavior, and those with higher self-efficacy in SHS avoidance behavior were more likely to practice such behavior [7]. Therefore, it is necessary to consider various internal factors, such as knowledge, attitude, and self-efficacy, when trying to identify factors affecting SHS avoidance behavior among Vietnamese adolescents.

SHS exposure commonly occurs at home, and it has been reported that among children and adolescents, it is higher in groups with lower economic level, when parents are smokers, and when parents have more positive attitudes toward SHS [8]. On the other hand, SHS avoidance behaviors were found to be higher when family members talked about the risks of SHS and did not ask their children to bring them, or light-up, their cigarettes [4]. Therefore, to prevent exposure to SHS and increase SHS avoidance behaviors, parents’ smoking status and attitude are important, and education within the household is influential.

According to previous studies, the risk of smoking among adolescents was higher when friends smoked, and the feeling of belonging to the peer group affected adolescents’ dangerous health behaviors [9,10]. Parents, on the other hand, played a protective role by helping their children to guard themselves against the influence of their friends. Children were encouraged to commit to healthy behaviors by their parents’ restrictions on undesired associations with peers who might negatively influence them and a good relationship with their parents [11]. As such, parents may have a positive effect on their children’s formation of relationships with their peers; however, it is difficult to exclude the influence of peer groups on health risk behaviors such as smoking and drinking. Moreover, like other oriental countries, Vietnam has a strong collectivistic culture [12]. Therefore, we included the role of both peer groups and parents in SHS avoidance behavior.

The stages of change model states that people move through five stages of change (pre-contemplation, contemplation, preparation, action, and maintenance) to carry out healthy behaviors. The pre-contemplation stage is a period of no intention to do certain things for six months. The contemplation stage is a period of intention to change for six months. The preparation stage is a period of intention to take action within 30 days. The action stage is a period of doing certain things for six months. Lastly, the maintenance stage is a period of doing certain things for over six months. People generally move across these stages in a nonlinear fashion [13]. In previous studies, the stages of change model was used to determine participants’ smoking behavior and intention to quit [14]. Our study also applied this model to investigate whether Vietnamese adolescents intend to change their SHS avoidance behavior.

According to WHO, majority of death caused by smoking would be occurred in economically disadvantaged countries within a decade [15]. However, most of studies regarding smoking hazards and SHS were conducted in well-developed parts of the world. While these studies have been increased recently in less developed countries [16,17], the study about SHS avoidance behavior has been limited in number. The Schwartz study [18] was one rare exception to this trend, but it focused on Canadian adolescents. Considering higher rates of smoking populations in less developed countries, it seems to be urgent to investigate the issue in these countries. Therefore, we carried out the research focusing on SHS avoidance behavior among Vietnamese adolescents. In the rural areas of Vietnam, the rate of SHS in adolescents is higher than that in urban areas. However, little research has been conducted on SHS avoidance behavior among adolescents in rural areas. Therefore, this study aimed to identify the stage of SHS avoidance behavior and related factors among adolescents in rural areas of Vietnam.

The purpose of this study was to examine the stage of SHS avoidance behavior of adolescents in rural areas of Vietnam and related factors affecting this behavior.

## 2. Materials and Methods

### 2.1. Research Design

This study used a cross-sectional research design to explore factors that may be associated with SHS avoidance behavior.

### 2.2. Participants

This study targeted adolescents in rural area of Vietnam. Adolescents were defined as second-year junior high school students. G*power 3.1.3 [19] as used to calculate estimated sample size, with significance level α = 0.05, medium effect size (OR = 1.72), power = 0.80 resulting in N = 177. As part of Official Development Assistance (ODA), Happy Quang Tri program, which was funded by the Korea International Cooperation Agency (KOICA), a survey was conducted with two communes; each commune has only one junior high school. Therefore, all 175 second-year junior high school students from the conveniently selected two communes in Quang Tri province, Vietnam, were recruited for this study. One adolescent’s parents disagreed to participate in this study, and eight incomplete responses were excluded. Finally, 166 samples were used for analysis.

### 2.3. Research Instrument

The questionnaire used in this study included items on general characteristics (gender, age; two items), frequency of SHS exposure (one item), stage of SHS avoidance behavior (one item), individual factors (belief about harm of SHS (one item), support for limiting exposure to SHS (one item), self-efficacy in SHS avoidance behavior (five items), refusal skills (five items), knowledge of tobacco hazards (ten items), and attitude towards smoking (twelve items)), interpersonal factors (presence of family members who smoke (one item), frequency of conversations with parents about smoking (one item), and sense of community with peer network (eight items)), making a total of 48 items. The questionnaire was developed in English, then translated into Vietnamese by a professor from a College of Health Sciences, and confirmed by a Vietnamese junior high school teacher with high English proficiency before use. Details of the research instrument are provided below.

#### 2.3.1. Frequency of SHS Exposure and Stage of SHS Avoidance Behavior

Frequency of SHS exposure was assessed with one item from the Youth Tobacco Survey (YTS) by the Centers for Disease Control and Prevention (CDC) [20]. The question “During the past seven days, how many days did you ride in a vehicle where someone was smoking?” was revised to “During the past seven days, how many days were you exposed to SHS?” to identify the level of secondhand smoke exposure in daily life. Response categories were zero days, 1 or 2 days, 3 or 4 days, 5 or 6 days, and seven days. To measure the stage of SHS avoidance behavior, an item developed by Schwartz et al. [18] based on the behavioral change phase model by Prochaska et al. [13] was used: “When exposed to SHS, do you continuously try to reduce this exposure?” Answers were categorized as pre-contemplation stage, contemplation stage, preparation stage, action stage, and maintenance stage. The definition of each stage was as follows: pre-contemplation stage—no intention of avoiding SHS within six months; contemplation stage—intention of avoiding SHS within six months; preparation stage—intention of avoiding SHS within 30 days; action stage—SHS avoidance behavior in the last six months; and maintenance stage—SHS avoidance behavior for over six months [13].

#### 2.3.2. Individual Factors

1. Belief about harm of SHS and support for limiting exposure to SHS. Items on belief about harm of SHS and support for limiting exposure to SHS were taken from the CDC YTS [20]. The question of belief about harm of SHS was as follows: “Do you think the smoke from other people’s cigarettes is harmful to you?” Belief about harm of SHS was rated on a four-point scale (1 = highly agree, 4 = highly disagree), with higher scores indicating beliefs that SHS is less harmful. The question of support for limiting exposure to SHS was as follows: “Which of these best describes what you think about smoking in indoor public places such as malls and restaurants?” Support for limiting exposure to SHS was rated on a three-point scale (1 = should never be allowed, 2 = can sometimes be allowed or in specific areas, 3 = should always be allowed).
2. Self-efficacy in SHS avoidance behavior. Self-efficacy in SHS avoidance behavior was assessed using five items, two of which referred to SHS avoidance behavior at home and on the street, and three items (when people come to my home to visit, at family holiday events, while participating in extracurricular activities) that were appropriate for the participants and were selected from a 13-item instrument developed by Li and Wang [21] based on the Self-efficacy to Resist Environmental Tobacco Smoke Exposure Scale (seven items) developed by Martinelli et al. [22]. A sample item of self-efficacy is as follows: “How confident you are that you can avoid other’s smoke when at home?” Each item was rated on a five-point scale (1 = not at all confident, 5 = extremely confident), with higher scores indicating higher self-efficacy in SHS avoidance behavior. Cronbach’s α was 0.83 in the study by Li and Wang [21] and 0.75 in this study.
3. Refusal skills. Refusal skills were measured using the Drug Refusal Skill Techniques (five items) developed by Epstein et al. [23]. A sample item of refusal skills is as follows: “Tell them not now.” Each item is rated on a five-point scale (1 = definitely would, 5 = definitely would not), with lower scores indicating better refusal skills. Cronbach’s α was 0.89 in the study by Epstein et al. [23] and 0.52 in this study.
4. Knowledge of tobacco hazards. Knowledge of tobacco hazards was measured using the instrument Knowledge of Tobacco Hazards (10 items) developed by Huang et al. [6]. A sample item of knowledge is as follows: “As long as we do not inhale secondhand smoke, smoking is not dangerous.” For each question, one point was given to correct answers, and zero to wrong or unsure answers. The total score ranged from 0 to 10, with higher scores indicating higher knowledge level. Cronbach’s α was 0.76 for the instrument at the time was development, 0.55 in this study.
5. Attitude toward smoking. Attitude toward smoking was measured using the Attitude toward Smoking instrument (12 items) developed by Huang et al. [6]. A sample item of attitude is as follows: “Smoking makes people happy.” Each item is rated on a four-point scale (1 = highly disagree, 4 = highly agree) and negative items are reversed when adding up the total score. Higher scores indicate a more favorable attitude toward smoking. Cronbach’s α was 0.73 for the instrument when developed [6], and 0.58 in this study.

#### 2.3.3. Interpersonal Factors

Family-related measures consisted of two items: presence of family members who smoke and frequency of conversation with parents about smoking, and regarding the peer group, eight items were used to assess sense of community with peer network.

1. Presence of family members who smoke and frequency of conversations with parents about smoking. Presence of family members who smoke and frequency of conversations with parents about smoking were measured using items from the CDC YTS [20]. The presence of family members who smoke is assessed using a yes/no item, and the frequency of conversations with parents about smoking using a five-point scale (1 = never, 5 = very often), with higher scores indicating more frequent conversations with parents about smoking.
2. Sense of community with peer network. Sense of community with peer network was measured using a revised instrument (8 items) developed by Nelson et al. [24] based on the Sense of Community scale by Peterson et al. [25]. A sample item of sense of community with peer network is as follows: “People in my network of friends are good at influencing each other.” Each item is rated on a five-point scale (1 = highly disagree, 5 = highly agree), with higher scores indicating a higher sense of community with the peer network. The reliability of the instrument was 0.92 at the time of development, 0.87 in the study by Nelson et al. [24], and 0.71 in the present study.

### 2.4. Data Collection

Data collection took place in January 2016, and the study was conducted after institutional review board approval (no. KBUIRB-201512-SB-031-02) from K-University. The researcher visited the participants’ junior high school and explained the reasons and purpose of the research to the principal and teacher, and after receiving approval, information letter and consent forms for parents were sent to the parents of all second-year students. The self-report questionnaire was distributed to the students who agreed to participate in the study. The average time for the students to complete the questionnaire was around 15 to 20 min. The written consent form included the purpose of the study, confidentiality of the data, benefits of participating in the study, and freedom to quit at any time during the study. The form was signed by hand.

### 2.5. Data Analysis

The collected data were analyzed using the following statistical methods with SPSS 21.0 (SPSS Inc., Chicago, IL, USA). Firstly, general characteristics of participants, SHS avoidance behavior-related characteristics, frequency of SHS exposure, and stage of SHS avoidance behavior were analyzed using descriptive statistics, such as frequency, percentage, mean, and standard deviation. Secondly, ordinal logistic regression was used to test for associations between factors and SHS avoidance behavior.

## 3. Results

### 3.1. Frequency of SHS Exposure and Stage of SHS Avoidance Behavior 

Participants were 80 male students (48.2%) and 86 female students (51.8%), of which 155 were aged 13 (93.4%), six aged 14 (3.6%), and five students offering no response (3%). As for the frequency of SHS exposure in one week, 60 students replied 1–2 days (36.1%), which was the highest number of respondents, 51 students replied 0 days (30.7%), 22 students replied 3–4 days (13.3%), 16 students replied seven days (9.6%), 14 students replied 5–6 days (8.4%), and three students provided no response (1.8%). As for the stage of SHS avoidance behavior, the maintenance stage had the highest number of respondents with 69 students (41.6%), 33 students were in the action stage (19.9%), 25 in the preparation stage (15.1%), 22 in the pre-contemplation stage (13.3%), and 17 in the contemplation stage (10.2%) (Table 1).

### 3.2. Individual and Interpersonal Factors

The smoking-related characteristics of Vietnamese adolescents were analyzed as individual and interpersonal characteristics. Regarding individual characteristic, 132 students (79.5%) answered that they strongly agreed that SHS was harmful, 27 students replied that they agreed (16.3%), four students strongly disagreed (2.4%), and one disagreed (0.6%) with that statement. A total of 142 students (85.5%) believed that indoor smoking should never be allowed in public spaces, and 21 students (12.7%) replied that it should be allowed in some areas. The mean score of self-efficacy in SHS avoidance behavior was 18.62 (range: 10–25), and that of refusal skills was 12.88 (range: 5–25). The mean score of knowledge of tobacco hazards and attitude toward smoking was 8.10 (range: 2–10) and 19.31 (range: 12–32), respectively. As for interpersonal factors, 79 students (47.6%) had family members who smoked, and 83 students (50%) did not have any family members who smoked. A total of 46 students (27.7%) answered that they “sometimes” had conversations with their parents about smoking, while 43 (25.9%) answered “often,” 25 (15.1%) “never,” 23 (13.9%) “rarely,” and 20 (12%) “very often.” The mean score of sense of community with peer network was 25.5 (range: 8–40) (Table 2).

### 3.3. SHS Avoidance Behavior According to Smoking-Related Characteristics

The results of the ordinal logistic regression analysis revealed that respondents who showed higher levels of self-efficacy and discussion with parents about the dangers of smoking tended to be at more advanced stages of SHS avoidance behavior. Although self-efficacy was statistically significant, the effect size of the variable was not quite high. Meanwhile, discussion with parents about the dangers of smoking had a higher effect size compared to self-efficacy (Table 3).

## 4. Discussion

Over 80% of tobacco deaths happen in low- and middle-income countries [15]. However, most studies regarding smoking and SHS were conducted in economically-advanced countries. Therefore, studies for SHS avoidance behavior using samples from less developed countries are greatly needed.

In this study, 67.5% of adolescents in rural areas in Vietnam experienced SHS more than one day a week, which was close to the rate for adults presented in data from 2010. It can be observed that relatively high rates of SHS are experienced by the rural Vietnamese participants in our study. Our result is also higher compared to the study with adolescents aged 12–15 in 68 low- and middle-income countries, which showed a total SHS rate of 55.9% [26].

In terms of the SHS avoidance behaviors of adolescents in rural areas of Vietnam, 41.6% of students in the study were in the maintenance stage, which involves maintaining a healthy lifestyle for more than six months [13]. The result of previous study [18] regarding SHS avoidance behavior showed that the proportion of maintenance was over 60% in four groups divided by frequency of SHS exposure. In this study, discussion with students’ parents regarding dangers of smoking was found to be significant factor influencing SHS avoidance behavior. Therefore, we assumed that a high percentage of maintenance was due to the parents’ influence. According to previous studies with adolescents, a tailored intervention program using telephone or computer was found to be effective in further securing the transition to and maintenance of the action stage [27,28]. Therefore, social network services and various multimedia should be utilized to help to maintain a healthy lifestyle among adolescents, and consideration should be given to the development of an intervention to enhance SHS avoidance behavior among Vietnamese youth.

The percentage of students in the pre-contemplation stage was about 13%. This is a stage where an individual does not feel the need for behavioral change and does not intend to change within six months [13]. According to Geraee et al. [29] and Kim et al. [30], individuals in the pre-contemplation stage moved on to the action stage after the provision of intervention programs, and according to Kim et al. [30] self-efficacy was the most important factor influencing behavioral change. Therefore, if an individual is in the pre-contemplation stage for a specific problematic behavior, it is necessary to motivate behavioral change by raising awareness of the behavior and increasing self-efficacy.

In this study, it was shown that among Vietnamese adolescents’ individual factors, self-efficacy in SHS avoidance behavior affected the stage of this behavior. This is consistent with previous studies on SHS avoidance behavior in adolescents [5]. Smorti [31] analyzed the relationship between health risk behaviors (dangerous driving, alcohol consumption, and cannabis use) and sensation-seeking and self-efficacy in Italian adolescents. The results showed that the level of health risk behaviors of adolescents was lower when the sensation seeking level was lower and self-efficacy against peer group pressure was higher. Therefore, self-efficacy in adolescents’ health behaviors is an important influencing factor, and strategies for enhancing it are needed to develop effective interventions. For example, one can allow adolescents to have successful experiences through simple tasks such as leaving places where one is exposed to secondhand smoke, or one might help increase adolescents’ self-confidence to avoid secondhand smoke by providing education. Lastly, another option could be the use of the role play method to deal with various situations that involve SHS exposure.

Previous studies have shown that individuals’ knowledge, attitudes, and beliefs have a significant impact on health-related behaviors. According to studies with adolescents, low levels of knowledge and positive attitudes toward smoking increased non-smokers’ intentions to smoke [32], but if smokers were provided with education at school and media about smoking cessation, the chances of smoking cessation increased [33]. Therefore, knowledge and attitudes toward smoking are important factors of smoking behavior among adolescents. However, in this study, beliefs and knowledge about smoking hazards, attitudes toward smoking, and refusal skills were not significantly associated with stages of SHS avoidance behavior. Gharaibeh et al. [34] analyzed the factors influencing SHS avoidance behavior among female non-smoking adults and found that knowledge and attitude about smoking hazards were high, but SHS avoidance behavior was low. Gharaibeh et al. [34] suggested that women’s cultural and gender role in Jordan could limit the practice of SHS avoidance behavior. Therefore, it is necessary to consider not only individual and interpersonal factors, but also socio-cultural factors when determining the factors influencing SHS avoidance behavior among Vietnamese adolescents.

As for family factors, more frequent conversations with parents about the harmfulness of smoking were associated with higher stages of SHS avoidance behavior. Dessie et al. [35] investigated factors limiting or enhancing communication with parents about reproductive health among Ethiopian adolescents and found that obstacles to the parent-child dialogue included “whether other people felt that the conversation with parents about reproductive health is not important” and “whether they thought their parents’ knowledge about reproductive health was low.” On the other hand, factors to increase conversation with parents included “whether they felt comfortable talking with their parents when they faced difficulties in their daily life” and “whether they watched TV with parents and talked about the contents.” Therefore, as various factors affect the frequency of health-related dialogue between parents and children, it is important to identify the obstacles to this dialogue and develop measures for the development of parent-participation programs. The results of this study suggest that exposure to SHS in households does not affect the stage of SHS avoidance behavior. However, in the study by Schwartz et al. [18], the stage of SHS avoidance in adolescents was lower when the exposure to SHS in the external environment including households was more frequent. When there was a family member who smoked, the frequency of SHS was higher and the perception of the risks of SHS exposure was likely to be lower. Therefore, in order to reduce the exposure to SHS of Vietnamese adolescents and increase avoidance behavior, it is necessary to promote smoking cessation among family members.

Adolescence is a time when peer influence is high, and peer groups influence health risk behaviors such as smoking and drinking. In this study, sense of community with the peer network did not affect the stage of SHS avoidance behavior; however, in a study by Nelson et al. [24] with adults over 18 years old, a higher sense of community with peer network led to higher practice of prevention behavior against sexually transmitted diseases. Therefore, the influence of the sense of community with the peer network should not be excluded as a preventive factor for health risk behaviors among adolescents. It is necessary to further analyze whether there is a difference in the degree of influence depending on age or type of health behavior.

In this study, individual and interpersonal factors were analyzed in relation to SHS avoidance behaviors. However, it is necessary to consider the influence of the local community on exposure to SHS. Nurwidya et al. [36] proposed policy measures to reduce smoking in Indonesia, including governmental policy intervention in tobacco advertising, policy on tobacco price, youth smoking prevention, support for smoking cessation, smoking restrictions in public places, and specifying non-smoker as an employee recruitment condition. Vietnam is one of the countries with the highest rates of smoking and SHS, and it is necessary to develop policies that can be practiced in this country to promote a healthy lifestyle among smokers and reduce the damage of SHS.

## 5. Conclusions

This study used a cross-sectional research design to explore the relationship between SHS avoidance behavior and associated factors among Vietnamese adolescents. In terms of the stage of SHS avoidance, the highest number of respondents were in the maintenance stage, followed by the action stage, preparation stage, pre-contemplation stage, and contemplation stage. Additionally, higher stages of SHS avoidance behaviors were associated with higher self-efficacy in SHS avoidance behavior, and more frequent conversations with parents about smoking. The results of this study are expected to be used as the basis for the development of SHS prevention programs for Vietnamese adolescents. For example, as discussion with parents about the dangers of smoking was the most important factor influencing SHS avoidance behavior, SHS prevention program involving parents needs to be considered to make parents play the role of educators at home. Despite the practical implications, this study also has some limitations. First, because this study investigated adolescents from two communes in Vietnam, the results cannot be generalized. Therefore, a randomized sample that includes wider areas would be necessary in future studies. Second, the reliability of the instruments assessing knowledge of smoking hazards, attitude toward smoking, and refusal skills was low. Therefore, a replication study on the factors influencing SHS avoidance behaviors in Vietnamese adolescents needs to be conducted using instruments with higher reliability measures. Third, as a result of baseline survey that was conducted eight months prior to the current study, only two students were current smokers. Therefore, we did not measure the percentage of smokers. However, the period could be long enough to change their smoking behavior, so the result of this study could be biased due to this factor. Therefore, it is suggested that some caution is needed in the interpretation of our results.

## Figures and Tables

**Table 1:** General characteristics (N = 166).

Variables	Categories	n	%
Gender	Male	80	48.2
	Female	86	51.8
Age (years)	13	155	93.4
	14	6	3.6
	No response	5	3
Frequency of SHS exposure	0 days	51	30.7
	1–2 days	60	36.1
	3–4 days	22	13.3
	5–6 days	14	8.4
	7 days	16	9.6
	No response	3	1.8
Stage of SHS avoidance behavior	Pre-contemplation	22	13.3
	Contemplation	17	10.2
	Preparation	25	15.1
	Action	33	19.9
	Maintenance	69	41.6

**Table 2:** Factors affecting the secondhand smoke avoidance behavior (N = 166).

Domain	Variables	Categories	n	%	Range	Mean ± SD
Individual	Belief about harms of SHS	Strongly agree	132	79.5		
		Agree	27	16.3		
		Disagree	1	0.6		
		Strongly disagree	4	2.4		
		No response	2	1.2		
	Support for limiting exposure to SHS	Never	142	85.5		
		Sometimes or some areas	21	12.7		
		No response	3	1.8		
	Self-efficacy of avoiding SHS				10–25	18.62 ± 3.99
	Refusal skill				5–25	12.88 ± 5.19
	Knowledge of tobacco hazards				2–10	8.10 ± 1.72
	Attitude toward smoking				12–32	19.31 ± 4.51
Interpersonal Family	SHS at home	Yes	79	47.6		
		No	83	50.0		
		No response	4	2.4		
	Discussion the dangers of smoking with parents	Never	25	15.1		
		Rarely	23	13.9		
		Sometimes	46	27.7		
		Often	43	25.9		
		Very often	20	12.0		
		No response	9	5.4		
Peer group	Sense of community with peer network				8–40	25.50 ± 6.77

SHS = Secondhand smoking.

**Table 3:** Stage to reduce exposure to the smoke by smoking characteristics (N = 166).

Domain	Variables	OR	95% CI	*p*
Individual	Belief about harms of SHS	0.751	0.399–1.412	0.374
	Support for limiting exposure to SHS	0.668	0.198–2.257	0.516
	Self-efficacy of avoiding SHS	1.170	1.054–1.300	0.003
	Refusal skill	1.011	0.934–1.093	0.794
	Knowledge of tobacco hazards	1.172	0.910–1.510	0.218
	Attitude toward smoking	1.010	0.922–1.107	0.829
Interpersonal Family	SHS at home	1.734	0.740–4.059	0.205
	Discussion the dangers of smoking with parents	1.932	1.329–2.807	0.001
Peer group	Sense of community with peer network	0.993	0.937–1.053	0.820

OR = Odds ratio; CI = Confidence interval.
